# Up-regulation of activation-induced cytidine deaminase and its strong expression in extra-germinal centres in IgG4-related disease

**DOI:** 10.1038/s41598-018-37404-x

**Published:** 2019-01-24

**Authors:** Yuka Gion, Mai Takeuchi, Rei Shibata, Katsuyoshi Takata, Tomoko Miyata-Takata, Yorihisa Orita, Tomoyasu Tachibana, Tadashi Yoshino, Yasuharu Sato

**Affiliations:** 1 Department of Pathology, Okayama University Graduate School of Medicine, Dentistry and Pharmaceutical Sciences, Okayama, Japan; 2 Division of Pathophysiology, Okayama University Graduate School of Health Sciences, Okayama, Japan; 3 Department of Pathology, Kurume University School of Medicine, Kurume, Japan; 4 Department of Otolaryngology, Okayama University Graduate School of Medicine, Dentistry and Pharmaceutical Sciences, Okayama, Japan; 5 Department of Otolaryngology, Himeji Red Cross Hospital, Hyogo, Japan

## Abstract

Immunoglobulin (Ig) G4-related disease (IgG4-RD) is a systemic disorder involving benign mass formation due to fibrosis and intense lymphoplasmacytosis; the chronic inflammation associated with the disease might also contribute to oncogenesis. Activation-induced cytidine deaminase (AID), normally expressed in germinal centre activated B-cells, is an enzyme that edits DNA/RNA and induces somatic hypermutation and Ig class switching. AID expression is strictly controlled under physiological conditions; however, chronic inflammation and some infectious agents induce its up-regulation. AID is overexpressed in various cancers and may be important in chronic inflammation-associated oncogenesis. We examined AID expression in IgG4-related sialadenitis (n = 14), sialolithiasis (non-specific inflammation, n = 13), and normal submandibular glands (n = 13) using immunohistochemistry and quantitative real-time polymerase chain reaction (qPCR). Immunohistochemistry revealed significantly more AID-expressing cells in IgG4-related sialadenitis than in sialolithiasis or normal submandibular gland samples (*P* = 0.02 and *P* < 0.01, respectively); qPCR yielded similar results. Thus, AID was significantly more up-regulated and had higher expression in extra-germinal centres in IgG4-RD than in non-specific inflammation or normal conditions. This report suggests that IgG4-RD has several specific causes of AID up-regulation in addition to inflammation. Furthermore, chronic inflammation-associated AID-mediated oncogenesis is possible in IgG4-RD.

## Introduction

Immunoglobulin (Ig) G4-related disease (IgG4-RD) is a systemic disorder characterized by the formation of benign masses and tumefactive lesions in various organs^1^. Histological observations of affected tissues exhibit dense fibrosis and infiltration of large numbers of lymphocytes, plasma cells, and eosinophils^2^. In patients with these types of diseases, many plasma cells express IgG4 and its serum levels are highly elevated^2^. The pathogenesis of IgG4-RD remains unclear; however, up-regulation of T-helper 2 (Th2) and regulatory T (Treg) cell cytokines, including interleukin 4 (IL4), IL5, IL10, IL13, and transforming growth factor-beta 1 (TGFB1), in affected organs is thought to be important^3^.

Patients with IgG4-RD appear to be at higher risk of developing malignancies than the general population; lymphomas and cancers of the colon, lung, breast, and pancreas, have been reported among those with this disease^4,5^. Moreover, the oncogenesis of extranodal marginal zone lymphomas, involving mucosa-associated lymphoid tissue (MALT lymphomas), has also been documented in patients with this disease^6,7^. Hence, IgG4-RD has been suggested to induce malignancies. Given the systemic inflammatory nature of this disorder, chronic inflammation may be important in such oncogenesis.

Activation-induced cytidine deaminase (AID) is a member of the cytidine deaminase family of enzymes that modify nucleotides to cause various mutations^8^. AID plays a crucial role in B-cell maturation and is normally expressed in activated B-cells in germinal centres^8^. This enzyme induces somatic hypermutations in the Ig variable region and gives rise to mutations in the constant region to cause Ig class switching from IgM to IgG, IgA, or IgE. Under normal conditions, AID expression is strictly regulated to prevent unfavourable somatic mutations. However, there have been many reports of inappropriate AID expression in various cancers^9,10^. In addition to B-cells, AID expression can be induced in various epithelial tissues due to chronic inflammation and infection^11^. Up-regulation of AID during inflammatory conditions is believed to cause uncontrolled somatic mutations of various genes, resulting in chronic inflammation-associated cancers^12^.

The mechanism underlying oncogenesis in IgG4-RD remains unclear, but AID up-regulation due to chronic inflammation could be a contributing factor. Despite this, AID expression in IgG4-RD has not been previously examined. Therefore, in this study, we investigated the expression of AID in IgG4-related sialadenitis.

## Results

### AID-expressing cells

**Figure 1:**
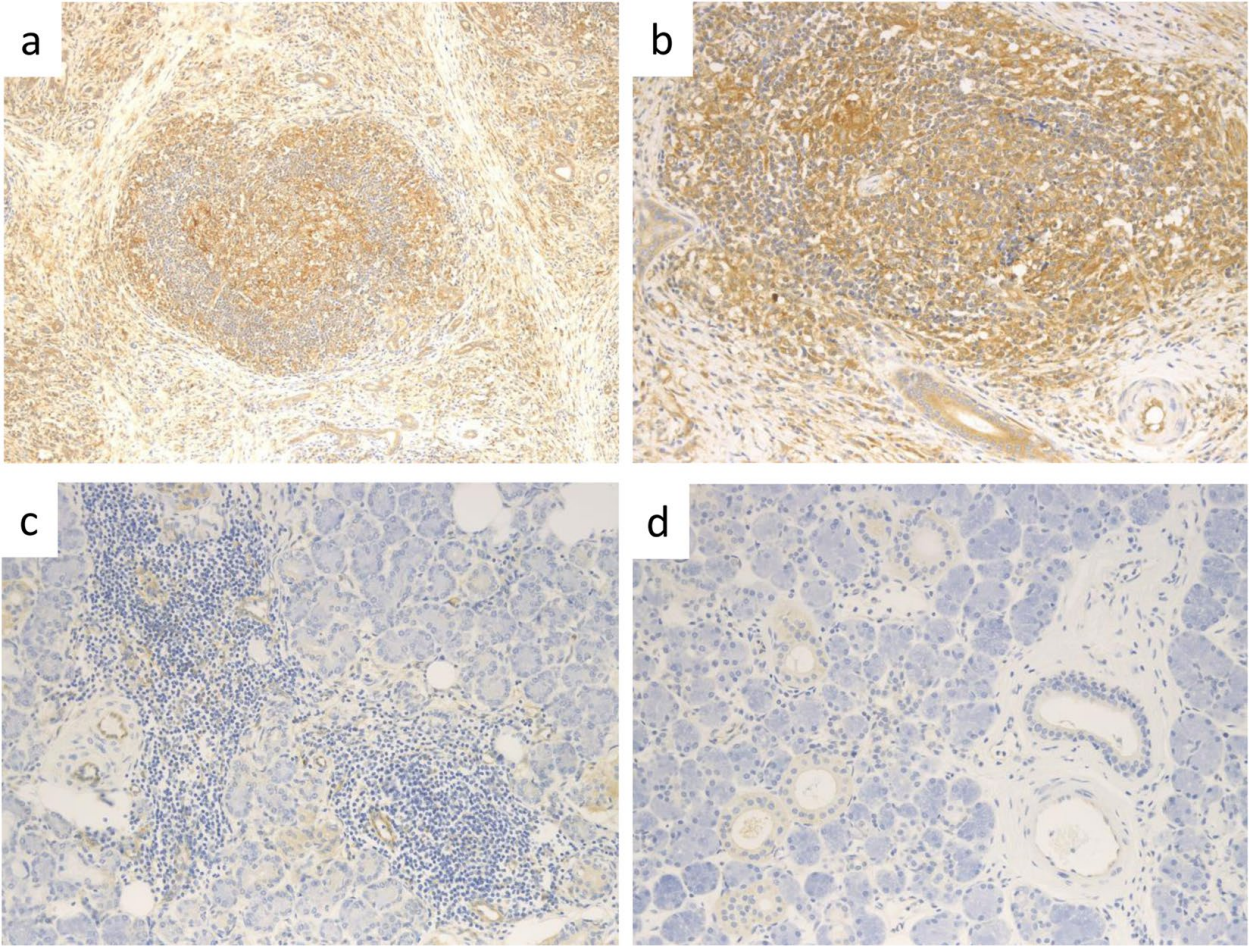
Immunohistochemical staining of activation-induced cytidine deaminase (AID). (**a**) IgG4-related sialadenitis (×100), (**b**) IgG4-related sialadenitis (×400). Greater numbers of strongly AID-positive cells were noted in IgG4-related sialadenitis than in those from healthy controls or patients with sialolithiasis. Ductal epithelia in IgG4-related sialadenitis were also positive for AID. In addition to AID-positivity in the germinal centres of AID-positive cells, the interfollicular areas were also AID-positive. AID-positive interfollicular areas were observed in lymphoid cells, plasma cells, and plasmacytoid cells. (**c**) Sialolithiasis (×400). Although lymphoid cell infiltration was observed in patients with sialolithiasis, fewer AID-positive cells were observed compared with IgG4-related sialadenitis. (**d**) Normal submandibular gland (×400). No AID-positive cells were noted in normal tissue.

**Figure 2:**
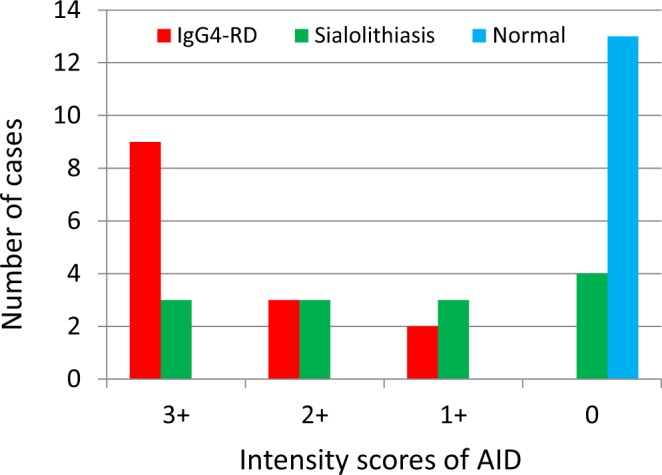
Intensity scores of specimens following immunohistochemical staining for activation-induced cytidine deaminase (AID). Compared with sialolithiasis and normal submandibular gland, AID was more strongly expressed in IgG4-related sialadenitis.

AID immunohistochemical staining revealed many AID-positive lymphoid or plasma cells in both the germinal centres and in the interfollicular areas of IgG4-related sialadenitis samples (Fig. 1a). The ductal epithelia in such specimens were also positive for AID (Fig. 1b). Various numbers of AID-expressing cells were observed in sialolithiasis specimens (Fig. 1c); however, none were noted in normal (control) tissues (Fig. 1d). The AID staining intensity index was significantly higher in IgG4-related sialadenitis samples [9 (64%), 3 (21%), 2 (14%), and 0 (0%) cases were categorized as 3+, 2+, 1+, and 0, respectively] than in sialolithiasis [3 (23%), 3 (23%), 3 (23%), and 4 (31%) cases, respectively] (*P* = 0.02) or normal control [all 13 (100%) cases were categorized as 0] (*P* < 0.01) (Fig. 2).

### Quantitative analysis of AID expression

**Figure 3:**
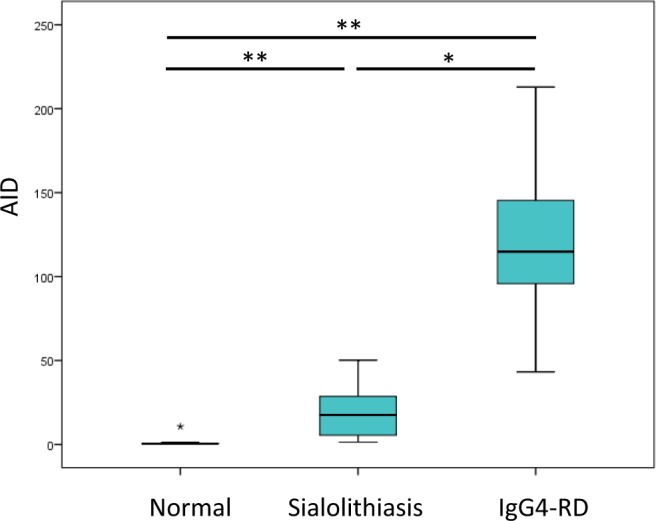
Quantitative analysis of activation-induced cytidine deaminase (AID) expression. AID expression in IgG4-related sialadenitis was significantly higher than in sialolithiasis and normal controls. *P < 0.05, **P < 0.01.

Quantitative real-time polymerase chain reaction (qPCR) revealed that *AID* expression in IgG4-RD was significantly higher than that in sialolithiasis (*P* = 0.02) or normal submandibular gland (*P* < 0.01) (Fig. 3). *AID* expression was also significantly higher in sialolithiasis than in normal control tissues (*P* < 0.01).

## Discussion

The aetiologies of IgG4-RD and its underlying oncogenic mechanisms remain largely unknown. Some studies have failed to find any relationship between IgG4-RD and cancer^13,14^, whereas others have shown malignancies develop in a subset of patients with IgG4-RD^15^. Hence, the relationship between the two remains controversial. However, the frequency of *KRAS* mutations has been reported to be higher in the pancreatic and bile ducts and gastrointestinal tracts of patients with autoimmune pancreatitis^16,17^. This indicates that IgG4-RD is associated with somatic oncogene mutations and may induce malignancies in various organs. The development of MALT lymphomas in patients with IgG4-RD has also been documented^6,7^. Interestingly, unlike conventional MALT lymphomas, those arising in patients with IgG4-RD are associated with up-regulation of Th2 and Treg cytokines^18^. Thus, these cytokines may be important in the pathogenesis of IgG4-RD; hence, MALT lymphoma may develop due to Th2/Treg-predominated chronic inflammation.

Various cancers occur in association with chronic inflammation. Infectious agents such as *Helicobacter pylori* and hepatitis B and C viruses are known to induce cancers related to chronic inflammation. Additionally, several non-infectious inflammatory conditions, such as inflammatory bowel disease, primary sclerosing cholangitis, and chronic pancreatitis, are also associated with malignancies^12^. The accumulation of genetic and epigenetic alterations, caused by chronic inflammation, is thought to be important in inflammation-associated oncogenesis. The majority of nucleotide alterations in cancers, including inflammation-associated cancers, involve cytosine (C)/guanine (G) to thymine (T)/adenine (A) transitions^19^. AID deaminates C residues to uracil (U), resulting in U/G mismatches. Without DNA repair, each U/G pair is replicated as T/A instead of C/G, representing the most frequent nucleotide transition observed in cancers. U/G mismatches can also be recognized by uracil-DNA glycosylase, which induces various mutations at such sites. In addition, the mutS homolog 2/mutS homolog 6 heterodimer can induce nucleotide mutations at both U/G and A/T sites near U/G mismatches. Hence, AID can induce several nucleotide sequence changes.

AID contributes to somatic hypermutation and Ig class switching under physiological conditions^20^. However, AID is also known to contribute to oncogenesis by inducing unfavourable somatic mutations and even chromosomal translocations. In hematopoietic malignancies, B-cell lymphomas with up-regulated AID have been shown to carry adverse mutations in genes, such as *MYC*, *PIM1*, and *PAX5*, as well as chromosomal abnormalities, including *MYC*/*IGH* rearrangements^12,21–23^. Aberrant AID expression in non-lymphoid cells has also been implicated in the development of malignancies^24^, with its up-regulation inducing somatic mutations in genes, such as *TP53*, *CDKN2A*, and *MYC*^24–27^. A previous report also described the relationship between AID expression and *IgH* rearrangements in the peripheral blood of patients with Mikulicz disease^28^.

In this study, AID was shown to be up-regulated in IgG4-related sialadenitis. AID expression was also elevated in sialolithiasis, but to a significantly lesser extent. These results indicate the existence of a mechanism other than inflammation leading to elevated AID levels in patients with IgG4-RD. Under physiological conditions, AID is up-regulated by the nuclear factor-kappa B (NF-κB) signalling pathway in a T-cell-dependent or -independent manner^29^. The NF-κB pathway plays a pivotal role in oncogenesis by inducing tumour cell proliferation and suppressing apoptosis. Infectious agents, such as hepatitis C virus and *H*. *pylori*, can induce NF-κB signalling to up-regulate AID^24,30^. Further, the NF-κB-dependent induction of proinflammatory cytokine expression, including that of tumour necrosis factor, is associated with AID overexpression^11^. In addition to these proinflammatory cytokines, the Th2/Treg cytokines IL-4, IL-13, and TGF-β are also known to result in elevated AID levels^12,28,31^. Further, Treg cytokines (IL-10 and TGFβ) and AID expression were significantly higher in the labial salivary glands of patients with IgG4-RD than in healthy controls or in those with Sjögren syndrome^32^. Hence, Th2/Treg-mediated immunity in IgG4-RD may be responsible for the observed up-regulation of AID.

Given the existence of germinal centre hyperplasia in IgG4-related sialadenitis, AID up-regulation may partially reflect increased numbers of germinal centres. However, histopathological examinations revealed many AID-positive lymphoid cells, plasma cells, and plasmacytoid cells in both the interfollicular areas and germinal centres of submandibular gland specimens from patients with sialadenitis, indicating broader aberrant AID expression. Thus, the Th2/Treg cytokines produced by interfollicular T-cells or other immune cells may cause such aberrant up-regulation.

## Conclusion

This study represents the first report of AID up-regulation and strong expression in extra-germinal centres in IgG4-RD. Thus, in this disease, AID may be up-regulated by Th2/Treg-predominated immunity and induce unfavourable somatic mutations in various organs. As a result, AID may play an important role in oncogenesis in patients with IgG4-RD.

## Methods

### Samples

Formalin-fixed paraffin-embedded tissues from patients with IgG4-related sialadenitis (14 cases), sialolithiasis (non-specific inflammation, 13 cases), and normal submandibular glands (13 cases) were examined. The median patient ages were 67.5 (range, 60–82), 42 (11–85), and 75 (49–92) years, respectively; the male/female ratios were 12/2, 8/5, and 12/1, respectively. This study was approved by the institutional ethical review board of Okayama University. Because the samples were limited to excess human tissue, the ethical review board waived the need for written consent from the patients.

### Histological examination and immunohistochemistry

All samples used in this study were surgically resected submandibular gland specimens. The specimens were fixed in 10% formaldehyde and embedded in paraffin, from which serial 3-μm-thick sections were cut and stained with haematoxylin and eosin. The sections were immunohistochemically stained, using an automated BOND-III stainer (Leica Biosystems, Wetzlar, Germany), using primary antibodies against AID (ab59361, polyclonal antibody, 1:150; Abcam, Cambridge, UK), IgG (A0423, polyclonal antibody, 1:20,000; DAKO, Glostrup, Denmark), and IgG4 (MC011, monoclonal antibody, 1:400; The Binding Site, Birmingham, UK).

### Confirmation of histological IgG4-RD diagnosis

All cases were reviewed by two pathologists. In accordance with the consensus statement for the pathological assessment of IgG4-RD, different high-power fields (eyepiece, 10×; lens, 40×) were examined to calculate the average number of IgG4-positive cells per field (>100) and the IgG4-/IgG-positive cell ratio (>40%)^33^. All 14 cases were histologically consistent with IgG4-related sialadenitis.

### Histological evaluation of AID expression

**Figure 4:**
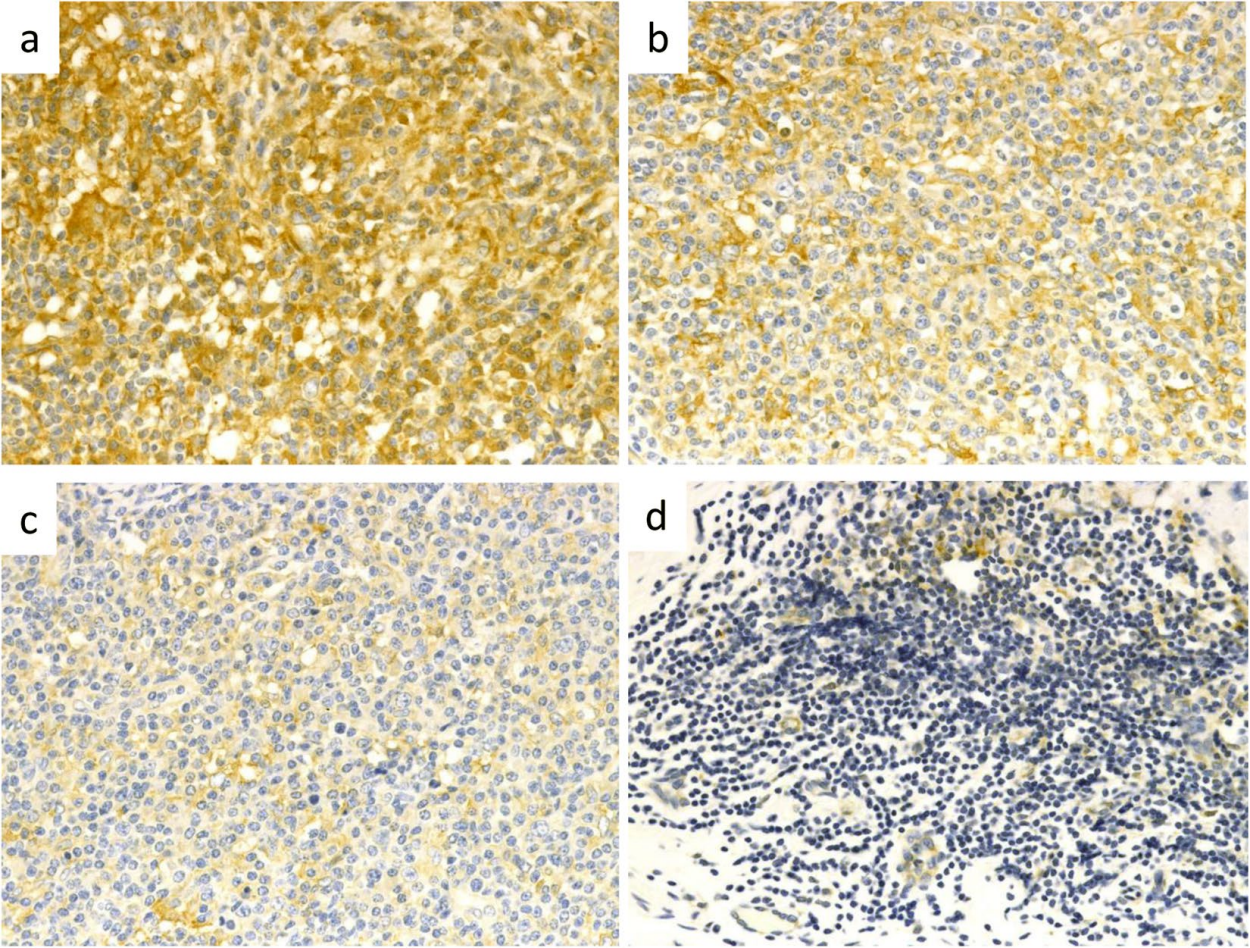
Intensity index of immunohistochemical staining for activation-induced cytidine deaminase (AID). (**a**) Strongly positive (3+), (**b**) moderately positive (2+), (**c**) weakly positive (1+), (**d**) negative (0). IgG4-related sialadenitis contained strongly AID-positive lymphoid and plasmacytoid cells in both the germinal centres and interfollicular areas (AID immunostaining, ×400 magnification).

AID positivity was evaluated based on an intensity index, with scores of 0 (negative), 1+ (weakly positive), 2+ (moderately positive), and 3+ (strongly positive) (Fig. 4). Each specimen was evaluated by two pathologists.

### qPCR

Total RNA was extracted from formalin-fixed, paraffin-embedded tissues with an miRNeasy FFPE kit (QIAGEN, Valencia, CA, USA), and complementary DNA was synthesized by reverse transcription PCR using a SuperScript VILO Master Mix kit (Invitrogen, Carlsbad, CA, USA), according to the manufacturers’ protocols. For quantitative analyses, multiplex real-time PCR was performed using TaqMan Gene Expression Assays (Applied Biosystems, Foster City, CA, USA) and a StepOnePlus Real-Time PCR System (Applied Biosystems), following the manufacturers’ instructions. Specific primers and probes for *AID* (Hs00757808_m1) and actin beta (*ACTB*; Hs01060665_g1) were obtained from Applied Biosystems. The PCR cycling conditions were: 2 min at 50 °C, 20 s at 95 °C, and 40 cycles of 1 s at 95 °C and 20 s at 60 °C. Expression of *AID* was normalized to that of *ACTB*.

### Statistical analysis

To compare AID expression among submandibular gland specimens from normal patients and those with IgG4-related sialadenitis or sialolithiasis, the Mann-Whitney *U* -test was applied (SPSS, version 24; IBM, Armonk, NY, USA). A *P*-value < 0.05 was considered statistically significant.
